# The effect of two different contemporary chelating agents on vital pulp therapy in mature permanent teeth with irreversible pulpitis using bioceramic material: randomized clinical trial

**DOI:** 10.1186/s12903-024-04627-6

**Published:** 2024-08-08

**Authors:** Yasmin Tawfik Mohamed Sobh, Mona Rizk Aboelwafa Ahmed

**Affiliations:** 1 Lecturer of Endodontics, Department of Endodontics, Faculty of Dentistry, Yasmin Tawfik Mohamed Sobh Lecturer of Endodontics, Sinai University - Kantara branch, Ismailia, 41636 Egypt; 2 Lecturer of Operative Dentistry, Department of operative Dentistry, Faculty of Dentistry, Sinai University - Kantara branch, Ismailia, 41636 Egypt

**Keywords:** Apple vinegar, Full pulpotomy, Postoperative pain, Visual analogue scale, Vital pulp therapy

## Abstract

**Background:**

Vital pulp therapy maintained functionality, vitality, and asymptomatic teeth. Compared to normal root canal treatment, pulpotomy was more helpful for irreversible pulpitis in adult permanent teeth. The research was aimed to assess effectiveness of vital pulp therapy using mineral trioxide aggregate with Apple Vinegar and Ethylene diamine tetra acetic acid (17%) for five minutes in adult carious exposed pulp of permanent teeth.

**Methods:**

Forty patients between 18 and 50 years old with a clinical diagnosis of symptomatic irreversible pulpitis but no periapical radiolucency were then divided randomly into two groups based on the irrigation method; ethylene diamine tetraacetic acid or apple vinegar. If pulpal bleeding could not be managed in less than six minutes, the assigned procedure was abandoned. After mineral trioxide aggregate application as a pulpotomy agent, glass ionomer and composite restoration were placed. Using a visual analogue scale, the pre and post-operative pain were recorded after 2,6,24,48, and 72 h. Success was assessed using radiographic and clinical examination data at three, six, and twelve months.

**Results:**

The success rate was discovered to be non-statistically significant in both groups after a year follow-up. Apple vinegar had a lower mean value than ethylene diamine tetra acetic acid at the preoperative baseline pain level, which was significant.Postoperatively, the ethylene diamine tetraacetic acid group reported the greatest mean value after two hours while Apple vinegar group reported the lowest mean values after 48 h (*P* < 0.05). After 72 h, pain level recorded insignificant difference.

**Conclusion:**

Apple vinegar yielded a marginally successful outcome but substantially improved pain alleviation.

**Trial registration:**

The trial was registered in Clinical trials.gov with this identifier NCT05970536 on 23/7/2023.

## Introduction

Vital pulp therapy (VPT) preserved tooth vitality, functionality, and asymptomatic state [1]. As a preventative strategy against more pulpal infection, critical mature permanent teeth with irreversible pulpitis underwent root canal therapy [2]. A well-executed root canal therapy causes a high success rate regardless of the technically challenging features and the loss of regenerative potential [3].

VPT procedures, including pulpotomy, were restricted to teeth in their immature state. More and more evidence has been shown that inflamed pulp tissue can recover “irreversibly,” independent of the maturity or immaturity of permanent teeth, provided that the pulpal inflammation is managed [2, 4].

Pulpotomy of closed apex permanent teeth with irreversible pulpitis offers advantages over traditional root canal treatment (RCT) such as simplicity, eliminating complications related to complex root canal architecture, maintenance of the proprioceptive sensation, and tissue regenerative potential period [5]. The potential advantages of bioceramic materials in endodontics are related to their physical, chemical, and biological properties. Bioceramic are biocompatible, non-toxic, non-shrinking, and usually chemically stable within the biological environment. A further advantage of these materials is their ability to form hydroxyapatite and ultimately create a bond between dentin and the material [6]. Bioceramic materials have antibacterial effect and comparable sealing ability. In terms of bioactivity and biocompatibility, these materials are able to induce hard tissue formation, i.e., osteogenesis, dentinogenesis, and cementogenesis. Based on such favorable properties, they are employed for VPTs with promising results [7, 8].

With advancements of biologically-based bioceramic materials, mineral trioxide aggregate (MTA) has gained focus in root repair [9]. It stimulated the undifferentiated cells recruitment and differentiation into odontoblast-like cells which enhanced dentinal bridge formation and pulpal healing [3]. Chelating agents remove the inorganic component of the smear layer. Chelation helps in dentin demineralization and the liberation of the essential growth factors present inside dentin. Acids also be used for the demineralization of dentin [10, 11].

Apple vinegar, a natural product. has been used in this study due to its antimicrobial effect, biocompatibility with cost effectiveness and its reported good efficiency in smear layer removal [12–14].

Chelating agents and disinfectants are essential for the successful outcome of endodontic treatment. Ethylene diamine tetraacetic acid (EDTA) has a considerable role in disinfection, smear layer removal, and dentinal erosion [15].

Apple vinegar is biocompatible and naturally produced through fermentation, which also results in higher acid contents of malic (0.35%) with acetic (5%) and citric acid 10% [16]. Citric acid has been used previously and showed acceptable results regarding stem cell viability and growth factor liberation [12].

Apple vinegar has antimicrobial properties and smear layer removal without detrimental impact on dentin interradicular calcium content [17].

Therefore, the effectiveness of VPT for mature permanent teeth with exposed carious pulp was assessed and compared in using a minimally invasive technique using MTA with two dissimilar chelating agents; Apple Vinegar and EDTA 17% for 5 min. The null hypothesis tested was that there was no effect of two distinct chelating agents on the success rate and pain assessment of VPT for mature permanent teeth with carious exposure.

## Methods

### Trial design

The trial was conducted as a randomized clinical study with two parallel groups that had the same allocation ratio and was planned as a double-blinded study including assessors and patients. ClinicalTrials.gov formally registered the trial protocol with the ID number (NCT05970536). The trial was conducted from December 2022 to December 2023 in the outpatient clinic at the faculty of Dentistry Kantara branch- Sinai University. In order to ensure clear and transparent reporting in this research, the CONSORT 2010 criteria (Consolidated Standards of Reporting Trial) were followed. Additionally, the Faculty of Dentistry and Cairo University’s Research Ethics Committee approved this trial (34/11/22). Before the study began, written informed consent forms were signed by each participant. For the volunteers’ ease of understanding, All consent form was created in Arabic. Participants were given a clear explanation of the trial’s objectives, benefits, risks, and anticipated duration in plain language.

### Eligibility criteria

Forty patients in the 18–50 age range who were in systemically good health were screened for trial enrollment. The chosen patients received training in dental hygiene along with a full mouth scaling procedure. Considering the study’s inclusion and exclusion criteria, a single session of treatment was conducted for each patient exhibited an exceedingly deep carious pulp exposure with a mature closed apex and normal apical tissue.

Following the completion of the history, clinical and radiographic examinations, as well as cold and electric pulp tests, were conducted to test for pulp sensibility; consequently, the diagnosis of periapical and pulpal tissue was established. Based on the history of persistent or unplanned pain was replicated by cold trials, a clinical diagnosis consistent with symptomatic irreversible pulpitis (SIP) was made (Endo-frost, Coltene, Whaledent GmbH). The results of inclusion showed that the teeth had a healthy periodontium, no radiographic evidence of periapical radiolucency, and no unfavorable reactions to percussion or palpation testing.

Exclusion criteria included patients taking analgesics during the last week, taking antibiotics within the last month, experiencing insufficient bleeding or being unable to control bleeding within five minutes of pulp exposure, pregnant females, or having medical issues and history of intolerance to anti-inflammatory drugs. While the teeth exclusion criteria were sinuses, swelling, a negative response to vitality test, non-restorable and had periodontal involvement or mobility, lacked pulp exposure or underdeveloped roots even after excavation of caries that had previously undergone treatment. Depending on clinical findings, patients who met the eligibility requirements were given information on VPT as well as the procedure’s risks and benefits. After that, signed informed consent was then acquired.

### Sample size calculation

MedCalc^®^ version 12.3.0.0 program “Ostend, Belgium” was used for calculations of sample size, statistical calculator based on 95% confidence interval and power of the study 80% with α error 5%, According to a previous study [18], showed that the percentage of perceptible grey discoloration in ProRoot MTA (80%) compared to Biodentine group (26.9%). So it can be relied upon in this study, based on this assumption, sample size was calculated according to these values produced a minimal samples size 28 sample were enough to find such a difference. So, by calculation, the sample size will be equal to 14 sample per group.

### Randomization

The entire pulpotomy will be finished up to the point where hemostasis can be easily obtained after pulp exposure has been clinically established. Subsequently, participants were randomized to the EDTA or apple vinegar groups. Using a Microsoft ^®^ Excel program, a computer-generated randomization was used to randomly allocate participants to each of the assigned treatments in the clinical trial. A list of sequential numbers was created, in which each randomly assigned participant in this list occupied a sequence number (ID) from “1 to 40” and was also given another randomized number represents either group 1 where the final irrigating solutions was 17% EDTA or group 2 where the final irrigation was apple vinegar. The generation of random allocation was done by Dr. (H R) who was not involved in this clinical trial and was independent from the recruitment process. The allocation sequence of the assigned participants in this clinical trial was kept with (H.R) in a sealed envelope which was known at the time the participant was given his informed consent to be involved in the clinical trial through contacting (H.R) by phone. The operator was not blind in to the study.

### Clinical procedure

Every patient had a pulpotomy procedure carried out by one operator utilizing aseptic measures and customary procedures. To achieve deep anesthesia, 2% lidocaine and 1:100 000 epinephrine (Ramson Remedies) was applied. Then, the tooth was sealed off with a rubber dam and cleaned with cotton pellets dipped in 5.25% sodium hypochlorite (NaOCl; Ammdent). Caries was removed with a sterilized big round diamond bur (ISO 001, Mani) and spoon excavators with high-speed hand pieces submerged in water coolant. A tapered round diamond bur was used to remove any residual supporting tooth structure. Composite resin (Fusion light-cured universal nanohybrid composite, Prevest DenPro), and sectional matrix (Saddle contoured metal matrices, Filaydent) were used to reconstruct missing proximal surfaces.

**Fig. 1:**
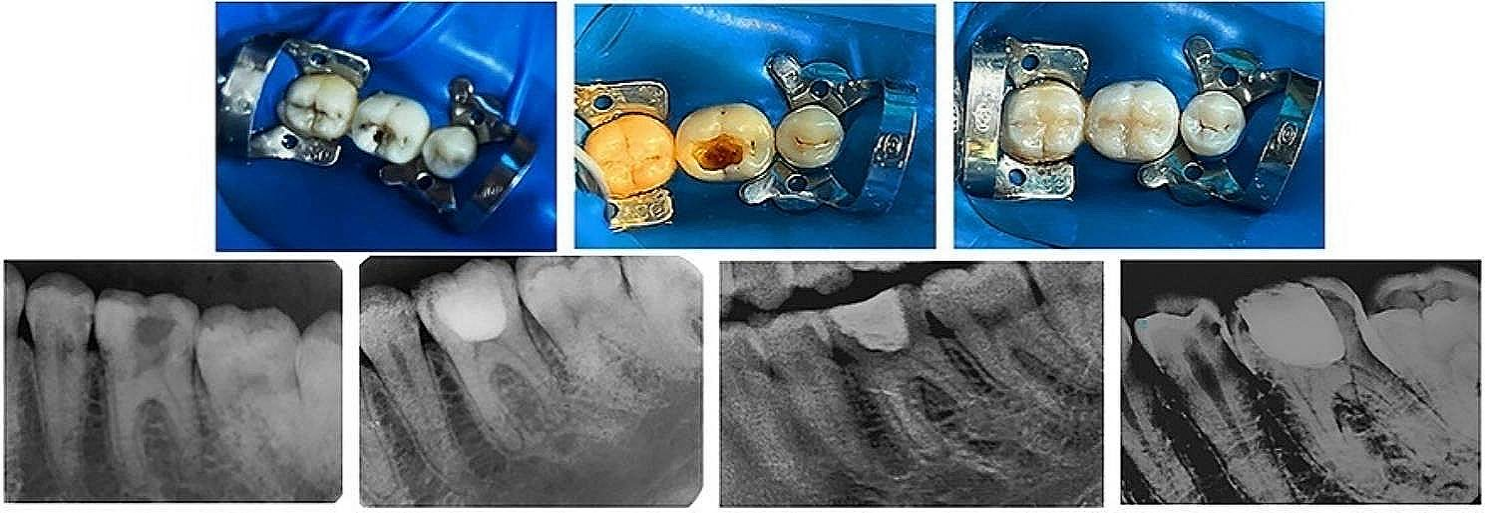
Clinical Photograph showed: Preoperative, full pulpotomy with hemorrhage control, Composite restoration. Clinical Radiographs showed: Preoperative, Postoperative at 3, 6 and 12 months, respectively

An aseptic tapered diamond bur was employed to refine the opening following pulp exposure. The vital pulp was clinically determined by the presence of pulpal bleeding. utilizing a water-cooled, high-speed hand piece with a sterile big round diamond bur, the exposed tissue of coronal pulp was gently removed Then, it was severed at the point where the root canal openings until hemostasis could be reached Fig. 1 Cotton pellets moistened in 3% NaOCl (Parcan, Septodont) were applied to the pulpal wound for a duration of two minutes, and up to five minutes if necessary till hemostasis occured.

RCT was used to handle all cases, regardless of the assigned group, where hemorrhage could not be halted within five minutes. Before the last treatment, the field was first cleansed with 5 ml of 1.5% NaOCl and then submerged in saline irrigation. Following hemostasis, the chosen teeth were randomly separated into two primary groups based on the final irrigating solutions: Group 1: A 17% EDTA for five minutes was used on exposed pulp tissue. Group 2: Apple vinegar was used to irrigate exposed pulp tissue for a duration of five minutes. According to the manufacturer’s recommendations, a bioceramic material ProRoot white MTA (Dentsply, DeTrey GmbH) was just mixed in both groups, transported to the pulpal wound utilizing an amalgam carrier, and gradually thinned to a thickness of 2–3 mm with dampened cotton granules to facilitate MTA settling, followed by a layer of glass ionomer as a temporary restoration for the coronal seal for some participants who have no time to stay for permanent composite restorations (GI; KetacTM Molar, 3 M Deutschland GmbH).

Consequently, the following day for those participants, the rubber dam was applied the cotton pellet and temporary restoration were withdrawn, and the MTA setting was verified. Afterwards MTA was covered with a thick coating of glass ionomer (KetacTM Molar, 3 M Deutschland GmbH) and composite resin as permanent restoration while for other participants permanent composite restorations were applied immediately over glass ionomer after vital pulp therapy procedures.

(Fusion light-cured universal nanohybrid composite, Prevest DenPro) was applied, polished, completed and corrected for occlusal contacts for all the included participants A Carestream RVG 5200 digital imaging system was used to take an instantaneous postoperative intraoral periapical radiograph (Carestream Health Inc.).

**Outcomes**The primary outcome revealed that the patients were returned back after three, six, and twelve months to evaluate the results. As recommended [19], the outcome assessment included both a clinical examination, a pain assessment and apulp response to a thermal and electrical pulp tester. Clinical evaluations were performed on teeth to determine whether any periapical or pulpal diseases were present, as well as their signs and symptoms. The pain assessment included patients that were told to utilize the 0–3 Visual Analogue Scale (VAS) to score their pain level [20]. Preoperative pain was documented by the patient at baseline, and postoperative pain was documented on VAS scores spaced out by 2, 6, 24, 48, and 72 h. There were four categories for pain scores: mild (Vas score 1), moderate (Vas score 2), severe (Vas score 3), and no pain (Vas score 0). The participants were instructed to go back to the clinician if their acute pain persisted even after taking the prescribed medication, and they were given instructions to take analgesics (Ibuprofen 400 mg every 6–8 h) for pain alleviation if necessary. Since ibuprofen has dose-dependent activity and its analgesic effect completely disappears after 8 h, these patients were evaluated at 24, 48 and 72 h similar to other patients in the study.

The secondary outcome showed the radiographic criteria which indicated absence of pathosis or resorption after three, six, and twelve months to evaluate the results. Lack of clinical and radiographic manifestations denoted that the treatment was deemed successful.

For statistical analysis, the statistical software for social sciences, version 23.0 (SPSS Inc., Chicago, Illinois, USA), had been used to evaluate the recorded data. An analysis of significance using paired samples t-test was performed when comparing related samples. ANOVA tests with repeated measures were used to see if there were any differences between related means. Bonferroni correction was applied in post-hock comparisons. To compare two means, the independent-samples t-test of significance was employed. Numbers and percentages were utilized to represent the qualitative characteristics. The Chi-square test and Fisher’s exact test were applied to compare groups with qualitative data. The likelihood, *P*-value was reported: *P*-value > 0.05 was deemed inconsequential, *P*-value < 0.05 was deemed noteworthy, and *P*-value < 0.001 was deemed extremely significant.

## Results

**Fig. 2:**
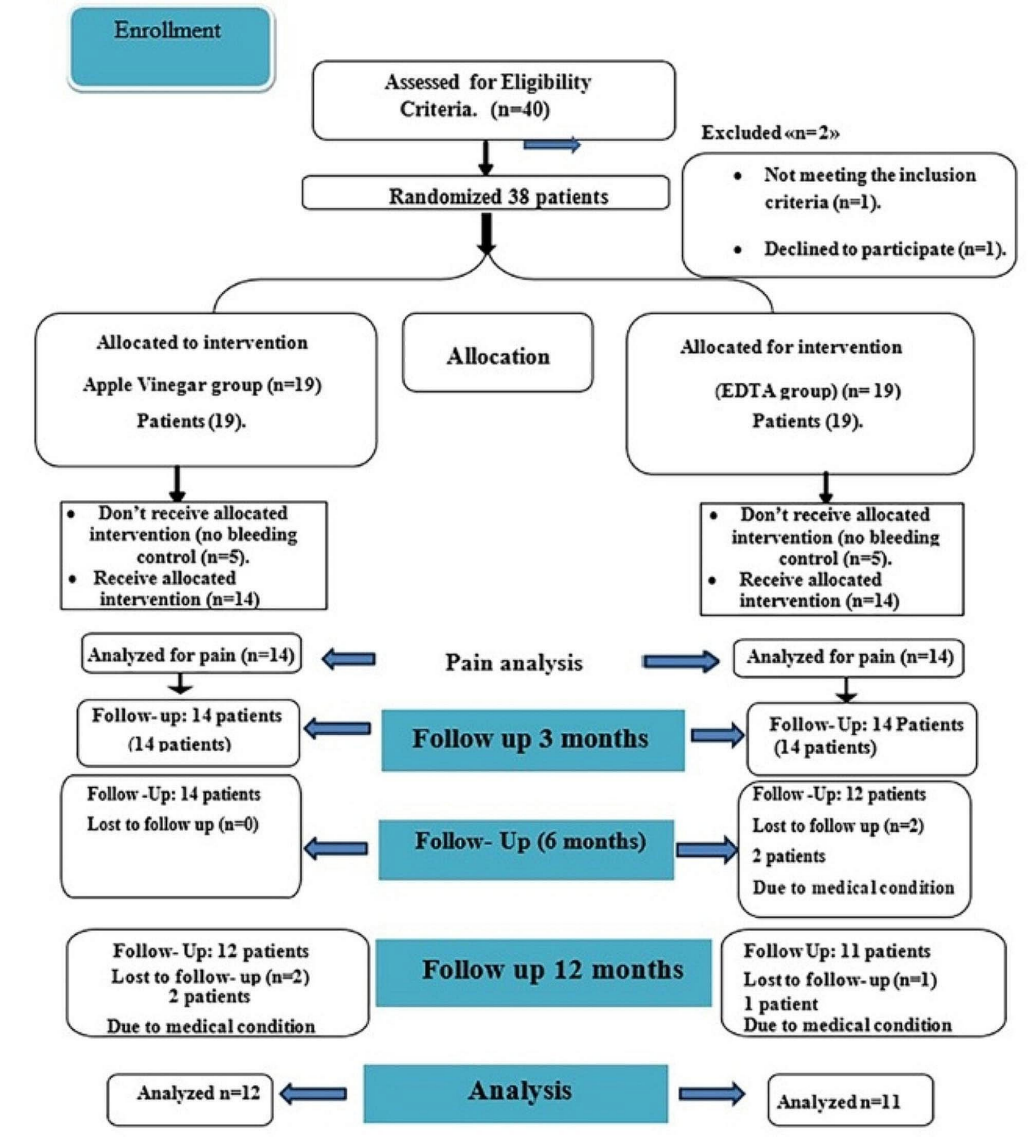
Photograph showed the Flow Chart of participants through trial

Regarding this research, 28 patients received the intervention. Because pulpal bleeding could not be stopped in the assigned period, the procedure could not be finished in 10 patients (5 in each group) Fig. 2 Age, gender, and tooth type did not significantly differ in mean values between the groups, according to demographic analysis (*p* > 0.05; Table 1).

**Table 1:** Analysis of the patients associating in the study

Baseline characteristics	EDTA Group (*n* = 14)	Apple vinegar Group (*n* = 14)	Test value	*p*-value	Sig
**Gender**					
Female	8 (57.1%)	10 (71.4%)	0.622	0.430	NS
Male	6 (42.9%)	4 (28.6%)			
**Age (years)**					
Mean ± SD	33.14 ± 9.34	33.14 ± 9.34	0.000	1.000	NS
Range	20–46	20–46			
**Tooth**					
Mean ± SD	32.86 ± 12.25	32.86 ± 12.25	0.000	1.000	NS
Range	16–46	16–46			

*Using: t-Independent Sample t-test for Mean ± SD;*

*Abbreviation: SD*,* standard deviation*

*NS: Non-significant* *P* *> 0.05*

Following year follow-up, the primary outcome demonstrated that the success percentage for the apple vinegar group was 78.6% (one case had clinical and radiographic failure, and two cases had not completed the follow-up) and for EDTA group was 57.1% (three cases had not completed the follow-up and three cases had clinical and radiographic failure). No statistically markble difference was presented between the two groups. (*p* > 0.05; Table 2).

**Table 2:** Comparison of success between the groups at 3,6,12 months follow up

Success of Radiographic	After 3 m	After 6 m	After 12 m	Test value	*p*-value
**EDTA Group** (***n*** = **14)**					
Success	9 (64.3%)	8 (57.1%)	8 (57.1%)	0.198	0.906
Failure	5 (35.7%)	6 (42.9%)	6 (42.9%)		
**Apple vinegar Group (** *n* * = 14)*					
Success	11 (78.6%)	13 (92.9%)	11 (78.6%)	1.371	0.504
Failure	3 (21.4%)	1 (7.1%)	3 (21.4%)		
**Chi-square test**	1.200	5.190	1.674		
**p-value**	0.549	0.158	0.433		

*p*-value > 0.05 is insignificant

Both groups, either EDTA or apple vinegar express less postoperative pain in comparison to the preoperative baseline .; however, the pain was significantly different in the pain level reduction over all time intervals, with the apple vinegar group showing a lower mean value than the EDTA group. Only the cases that got therapy and follow-up were included in the secondary outcome for pain evaluation. The pain assessment was recommended for all patients up to 72 h after the intervention period. A remarkable decrease in pain was noticed across the entire time intervals in the intragroup comparison, with a meaningful difference for both groups (*p* < 0.05; Table 3). The EDTA group reported the highest mean value after two hours, while the apple vinegar group reported the lowest mean value after 48 h (*p* < 0.05). At 72 h, there was no significant difference (*p* = 0.060). After 72 h, no patients in either group reported bearable to extreme discomfort (Table 4). At the conclusion of the trial, twenty-three patients were evaluated clinically and radiographically (two patients in Apple vinegar category and three patients in the EDTA category were gone during follow-up). Three teeth in the EDTA category and one tooth in the apple vinegar category were classified as having an acute clinical breakdown because the patient experienced intense impetuous pain that persisted for several days following the intervention and showed symptoms of radiographic pathosis, RCT had been done in such cases.

**Table 3:** Pre- and post-operative pain intensity among tested groups at different time intervals

VAS score Pain	EDTA Group (*n* = 14)			Apple vinegar Group (*n* = 14)		
	Mean ± SD	MD from Pre	*p*-value	Mean ± SD	MD from Pre	*p*-value
Pre-operative	2.43 ± 0.85	Ref.		2.43 ± 0.85	Ref.	
After 2 h.	2.82 ± 0.40	0.39	0.244	1.92 ± 0.90	-0.51	0.020*
After 6 h.	2.73 ± 0.47	0.30	0.166	1.33 ± 1.15	-1.10	0.018*
After 24 h.	2.00 ± 0.89	-0.43	0.032*	0.83 ± 1.03	-1.60	0.010*
After 48 h.	1.55 ± 0.93	-0.88	0.018*	0.50 ± 0.90	-1.93	0.008*
After 72 h.	1.00 ± 1.34	-1.43	0.005*	0.25 ± 0.87	-2.18	< 0.001**

*p-value < 0.05 is significant; **p-value < 0.001 is highly significant

**Table 4:** Comparison of post-operative pain intensity according to VAS score Pain in the tested groups at 2, 6, 24, 48, and 72 h

Pain (VAS score)	EDTA Group (*n* = 14)	Apple vinegar Group (*n* = 14)	t-test	*p*-value	Sig.
After 2 h	2.82 ± 0.40	1.92 ± 0.90	3.047	0.006	S
After 6 h	2.73 ± 0.47	1.33 ± 1.15	3.728	0.001	HS
After 24 h	2.00 ± 0.89	0.83 ± 1.03	2.888	0.009	S
After 48 h	1.55 ± 0.93	0.50 ± 0.90	2.726	0.013	S
After 72 h	1.00 ± 1.34	0.25 ± 0.87	1.607	0.123	NS
*p*-value	< 0.001**	< 0.001**			

NS: Non-significant; (*p* > 0.05)

S: Significant; (*p* < 0.05)

HS: Highly significant; **p-value < 0.001

## Discussion

Clinical practice revealed many cases of carious pulp exposure [21]. RCT was the usual therapy for teeth suffering from irreversible pulpitis in the past. In addition to the brittleness of the surviving structure and the loss of their ability for regeneration, the process is invaisive and technically difficult [3]. Thus, VPT should be viewed as a conservative and less invading substitute for root canal therapy. Numerous studies assessed the effectiveness of a complete pulpotomy as the radicular pulp reversibly inflamed while the coronal pulp showed irreversible inflammation, making the histological diagnosis unclear [22]. VPT overall success in treating cariously exposed teeth depends on a number of factors, including the pulp tissue’s inflammatory state, the length of the observation period, the success criteria, and the pulp therapy agent’s biocompatibility [23].

Research on the application of calcium hydroxide in clinical settings has demonstrated adverse effects on pulp viability [24]. Therefore, researchers have proposed MTA as a substitute due to its good outcomes and odontogenic action [25]. To avoid being contaminated by microorganisms or the possibility of pain over many visits, this study only involved vital pulp therapy procedures in one visit [26].

In the current trial; there is a deep anesthetic effect and a 15-minute pause before starting the procedure to allow the nerve induction to get blocked [27]. Two distinct chelating chemicals, namely EDTA 17% and apple vinegar, will be compared with MTA to assess the efficacy of VPT for permanent teeth with a closed apex that have exposed carious pulp. Every tooth in the study had clinical indications of SIP, as determined by the diagnostic. In addition, as a crucial first step, the irrigating techniques should support the stem cells’ proliferation and differentiation while also preserving their viability [28].

Elevated NaOCl concentrations significantly impair stem cell apical papilla ability to survive and differentiate. Nevertheless, this impact can be avoided by using 1.5% NaOCl and then 17% EDTA to detach the inorganic smear layer component, demineralize dentin, bedside release of growth factors trapped inside root dentin [10–29]. Apple vinegar is a natural solution made of 10% citric acid, 0.35% malic acid, and 5% acetic acid. It is created through fermentation. It was said to be effective in removing smear layers and to have antibacterial properties, biocompatibility, and cost effectiveness [12].

The current study’s findings demonstrated that, when utilizing both EDTA and apple vinegar following a full pulpotomy, age had no discernible impact on the VPT prognosis with MTA. It was consistent with earlier research [3, 30–32], which may have been caused by the study’s narrow age range. However, it is not in line with previous published research [33].

A number of techniques were proposed to manage pulpal bleeding [34]. If the bleeding does not stop after five minutes, the pulpotomy is abandoned, and the case is declared unsuccessful because the inflamed pulp has spread to the radicular pulp, necessitating more pulp tissue removal [35]. Thus, in our study, a corresponding technique is tried in carefully chosen examples. Regarding full pulpotomy, neither group showed a statistically substantial variation in success rate. By creating paths for microleakage, the state of the restoration and periodontal health played a role in the pulpotomized teeth’s failure [31].

Despite the use of MTA and an overlaying dual layer of restoration, the three failed teeth in the current investigation had perfect restorations with good periodontium at the moment of failure. One possible explanation for these shortcomings could be the inability to accurately ascertain the accurate preoperative pulpal inflammatory condition. Another example of a failure in the Apple vinegar group occurred when the fracture line extended to exposed pulp resulting in fragmented restorations coronally at the conclusion of the follow-up. The present investigation revealed that both groups experienced overall success, with the apple vinegar group achieving a better success rate than the EDTA group across each time interval, however not statistically significant (*p* > 0.05). These findings were linked to calcium ion which apple vinegar releases from dentin, which increases the synthesis of fibronectin, a protein that is crucial for cell adhesion [12]. Furthermore, a prior study found that dentin is demineralized by chelation with 17% EDTA and decalcification with apple vinegar [36]. It is also noteworthy that 10% citric acid in apple vinegar and 17% EDTA expose a stable collagen matrix with respectable results for the release of growth factors and the viability of stem cells [37]. These findings were consistent with earlier research [12&38].

These could also be attributed to the better healing capacity of apple vinegar which was in agreement with other reported studies [13, 14]In the current investigation, VAS was selected because it is a more dependable, straightforward, sensitive, and easy way to assess pain [39]. Preoperative baseline data on pain intensity was collected, and postoperative data was fetched at various intervals. Since the anesthetic solution fades off after six hours and post-operative discomfort occurs mostly after 12, 24, and 48 h, that was the chosen time [40].

Complete pain relief following a full pulpotomy was reported as occurring non-significantly after 72 h, and there was a notable distinction in post-operative pain for both groups up to 48 h after the procedure, with the lowest mean value of post-operative pain occurring after 48 h and the highest mean value occurring after 2 h. In the early postoperative days, there was a substantial difference between time intervals and preoperative pain, indicating pulpal inflammation and high preoperative pain levels.

These outcomes were attributed to the endodontic treatment-induced escalation of the reaction of inflammation in the periapical tissues. Within the first few hours of the injury, polymorphonuclear leukocytes (PMN) invaded the damaged area, which was followed by the rise of inflammatory mediators’ release [41]. On the other hand, the inflammatory reactions initiated by the bacterial invasion causing preoperative pain last from several minutes to several days .In pulpotomy there was cutting the terminal ends of nociceptive sensory neurons, the reduction in local tissue pressure and inflammatory mediator concentrations in pulpotomy treatment may affect postoperative pain intensity [3]. After 48 h, the proliferative process started, which was marked by a fast fall in inflammation and a decrease in the PMN population throughout the first two days, along with a decrease in pulp pressure [42]Therefore, pulpotomy treatment may affect the postoperative pain intensity and pulpotomy has proven to be more effective in relieving pain [3].

Compared to the EDTA group, apple vinegar produced improved pain management. This could be explained by the anti-inflammatory properties of malic acid, which was found in apple vinegar and had been linked to a decrease in pain [43]. These findings are consistent with other reported studies [44, 45]. Pulp sensitivity was unreliable due to the possibility of false-negative responses from deep-seated coronal restorations in complete pulpotomies and increased pulp distance.

From the limitations of the study; the assessment of late failures like pulp canal obliteration, future studies with a wider age range, classifying cases of uncontrollable bleeding as failures and long follow-ups are beneficial for assessing the hard tissue barrier, especially with newer, more precise tools like cone-beam computed tomography.

## Conclusions

Apple vinegar yielded non significantly successful outcome but substantially improved pain alleviation; therefore apple vinegar generated a positive result on postoperative pain for VPT of mature permanent teeth with carious exposure so the tested hypothesis was rejected, while regarding the success rate, it was approved.

## Data Availability

Upon request, the corresponding author will provide the data supporting the study’s conclusions. Due to [restrictions, such as its having information that could threaten the privacy of research participants], the data are not publicly available.

## Declarations

### Competing of interest

No competing of interest.

## Abbreviations

VPT: Vital pulp therapy
EDTA: Ethylene diamine tetra acetic acid
RCT: Root canal treatment
VAS: Visual analogue scale
MTA: Mineral trioxide aggregate
SIP: Symptomatic irreversible pulpitis
NaOCl: Sodium hypochlorite
PMN: Polymorphonuclear leukocytes
